# Neuroprotective Effect of Bioactive OC1 Peptide on Alzheimer's Disease Cell Model Generated by Aβ_25‐35_ Induced PC‐12 Cell Line

**DOI:** 10.1002/alz70855_101906

**Published:** 2025-12-23

**Authors:** Zeynep Didar Kayıklık, Bilal Çakır, İbrahim Gülseren, Zeynep Büşra Bolat

**Affiliations:** ^1^ Health Science University, İSTANBUL, Turkey, Turkey; ^2^ İstanbul Sabahattin Zaim University, İstanbul, Turkey, Turkey

## Abstract

**Background:**

Alzheimer's disease is associated with dementia and occurs due to the misfolding and extracellular accumulation of amyloid‐beta (Aβ) protein in the brain. Aβ_25‐35_ peptide is a toxic form of Aβ commonly used in in vitro models. Although no definitive cure exists, several FDA‐approved drugs help patients maintain mental function. Bioactive peptides are specific protein fragments and are distinguished by their ability to promote positive effects on different neurodegenerative diseases. Bioactive peptides derived from hazelnuts (*Corylus avellana* L.) hold promise for neuroprotective effects against Alzheimer's disease.

**Method:**

Alzheimer's disease model was established using PC‐12 cell line treated with Aβ_25‐35_. Toxic doses for the disease model were determined using resazurin‐based cell viability assays. Subsequently, bioactive peptide derived from hazelnuts were applied to the Alzheimer's disease model, and the neuroprotective effect doses were identified through cell viability assays. Colony forming unit assay was performed to analyze the growth inhibition on Alzheimer's disease model. Cell cycle and Annexin V/PI assay was evaluated using flow cytometry. 2′,7′‐dichlorofluorescin diacetate (DCF‐DA) reagent was used to detect intracellular ROS production by fluorescent microscopy.

**Result:**

OC1 bioactive peptide from hazelnut ameliorated the cell viability of *in vitro* Alzheimer's model. OC1 peptide was further evaluated for their neuroprotective effects at the molecular level. Cell cycle analysis revealed that Alzheimer's model group (Aβ_25‐35_ treated) showed G2/M phase arrest in cell cycle assay when compared to control. Conversely OC1 peptide treatment improved the G2/M arrest caused by Aβ_25‐35._ Annexin V/PI results demonstrate that Aβ_25‐35_ treated group induced late apoptosis, while OC1 peptide treatment reversed this effect. OC1 peptide exerted neuroprotective effect by reducing intracellular ROS formation compared to Aβ_25‐35_ treated alone.

**Conclusion:**

Our findings indicate that the newly synthesized bioactive peptide OC1 is promising as potential neuroprotective agents for neurodegenerative diseases and may offer encouraging results for the prevention of Alzheimer's disease.

This study was supported by Türkiye Sağlık Enstitüleri Başkanlığı, grant number: 22740 TÜSEB. Attendance at this conference was supported by an AAIC 2025 Conference Fellowship.

